# Long-term outcomes of a randomized controlled trial on brief alcohol interventions for job-seekers

**DOI:** 10.1186/1940-0640-8-S1-A69

**Published:** 2013-09-04

**Authors:** Inga Schnuerer, Sophie Baumann, Beate Gaertner, Katja Haberecht, Gallus Bischof, Ulrich John, Jennis Freyer-Adam

**Affiliations:** 1 Institute of Epidemiology and Social Medicine, University Medicine Greifswald, Greifswald, Germany; 2 Institute of Biometry and Clinical Epidemiology, Charité University Medicine Berlin, Berlin, Germany; 3 Department of Epidemiology and Health Monitoring, Robert Koch Institute, Berlin, Germany; 4 Department of Psychiatry and Psychotherapy, Medical University of Lübeck, Lübeck, Germany

## 

In the past three decades, two paths of how to understand and support health behavior change have been followed: stage-tailored models and continuum models. Our aim was to investigate the comparative efficacy of a stage-tailored intervention and a non-stage-tailored intervention of the same intensity in reducing alcohol use and alcohol problem severity among job-seekers with unhealthy alcohol use. The interventions were based on the most prominent representative models, the transtheoretical model of behavior change (TTM) and the theory of planned behavior (TPB). A total of 1,243 proactively recruited job-seekers with unhealthy alcohol use (64% male, M=30 years, 76% not motivated to change) were assigned by random to three study groups: (1) individualized stage-tailored feedback letters based on the TTM, (2) individualized non-stage-tailored feedback letters based on the TPB, and (3) assessment only (controls). Both intervention groups received up to two interventions. All groups were followed-up three, six and 15 months after baseline. Piecewise latent growth models were conducted to investigate change in alcohol use and severity from baseline to month 3 and month 3 to 15. Initial motivation was tested as a moderator of intervention efficacy. All groups reduced alcohol use and severity significantly in the short term. In the long term and when including motivation as a covariate, the stage-tailored group produced a significantly greater reduction than the two other groups. Initial motivation was a significant moderator of intervention efficacy. Neither intervention was generally more or less effective than the other. The efficacy of the interventions largely depended upon the initial motivation to change alcohol use: the lower the motivation to change, the more likely the individuals were to benefit from the stage-tailored intervention; and the higher the motivation to change, the more likely the individuals were to benefit from the non-stage-tailored intervention.

